# Critical Aspects of Person Counting and Density Estimation

**DOI:** 10.3390/jimaging7020021

**Published:** 2021-01-31

**Authors:** Roland Perko, Manfred Klopschitz, Alexander Almer, Peter M. Roth

**Affiliations:** 1 Joanneum Research Forschungsgesellschaft mbH, DIGITAL, Remote Sensing and Geoinformation, 8010 Graz, Austria; manfred.klopschitz@joanneum.at (M.K.); alexander.almer@joanneum.at (A.A.); 2 Data Science in Earth Observation, Technical University of Munich, 82024 Taufkirchen, Ottobrunn, Germany; peter.roth@tum.de

**Keywords:** person counting, density estimation, convolutional neural network, deep learning

## Abstract

Many scientific studies deal with person counting and density estimation from single images. Recently, convolutional neural networks (CNNs) have been applied for these tasks. Even though often better results are reported, it is often not clear where the improvements are resulting from, and if the proposed approaches would generalize. Thus, the main goal of this paper was to identify the critical aspects of these tasks and to show how these limit state-of-the-art approaches. Based on these findings, we show how to mitigate these limitations. To this end, we implemented a CNN-based baseline approach, which we extended to deal with identified problems. These include the discovery of bias in the reference data sets, ambiguity in ground truth generation, and mismatching of evaluation metrics w.r.t. the training loss function. The experimental results show that our modifications allow for significantly outperforming the baseline in terms of the accuracy of person counts and density estimation. In this way, we get a deeper understanding of CNN-based person density estimation beyond the network architecture. Furthermore, our insights would allow to advance the field of person density estimation in general by highlighting current limitations in the evaluation protocols.

## 1. Introduction

**Figure 1:**
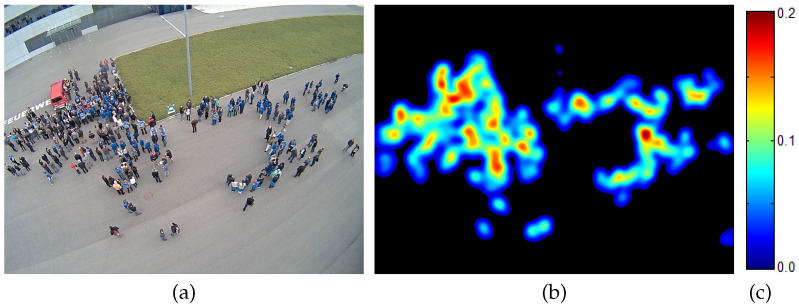
Person counting via density estimation. (**a**) Input image, (**b**) estimated density, and (**c**) colorbar that defines the color mapping to the fractional human count. For this example the true human count equals 303, while the given density predicts 294. Example adapted from our paper [8].

**Figure 2:**
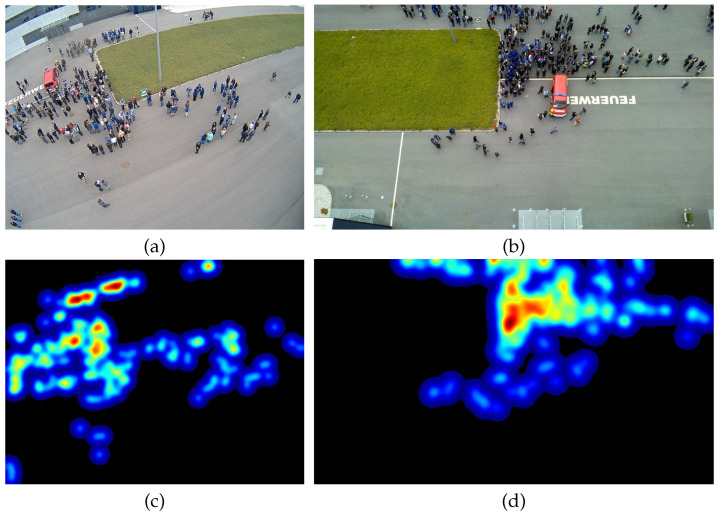
Both images in the top row (**a**,**b**) contain 254 people, however, the densities in the bottom row (**c**,**d**) have different spatial distributions.

Person counting and density estimation from single images is an active field of research in computer vision, artificial intelligence, and machine learning. In general, density estimation is defined as to calculate the human density of a given input image and thus the number of persons in the image (person count or human count). The density itself holds the number of persons for each pixel which is mostly a fraction since one human occupies a region and thus many pixels. An example is given in Figure 1. In addition, as can be seen in Figure 2, the density map holds more information than just the human count, as also their spatial distribution is revealed.

While the derivation of a human or, in general, an object density from a given input image could just be seen as a scientific challenge, there are real-life applications. As known from the literature, the recognition of critical situations in crowded scenes is very important to prevent escalations and human casualties [1,2,3]. On large scale events, like music festivals, sports events, or demonstrations, there are a few important parameters to define the riskiness of a situation. According to [4] the number of persons, the density of individuals per square meter, the general motion direction of groups, and individual motion patterns have main impacts. Especially dangerous forward and backward motion in front of a region of interest, like a stage or entrance, has to be taken into account. These parameters can be used to estimate the so-called *human pressure* which indicates potential locations of violent crowd dynamics [5]. Human pressure is defined as

(1) P(x,t)=ρ(x,t)Var(V(x,t)),

with x the spatial location, *t* the time, ρ the estimated density, and *V* the motion. The rationale behind this measure is that a high human density (i.e., large density ρ(x,t)) is not dangerous per se, however if those persons move in different direction or in stop-and-go waves (i.e., large velocity variation Var(V(x,t))) then the situation becomes critical. Pressures exceeding the value of $0.02/s^{2}$ indicate turbulent crowd dynamics, which was empirically determined in [5]. Thus, the measure of human pressure can be used to identify the beginning of turbulent crowd dynamics which allows releasing counter-actions to calm the situation before people get injured. Under certain constraints computer vision technology can be used to derive this human pressure and systems for doing so were, for instance, presented in [3,6,7]. The main components are a video camera to grab images, an algorithm to estimate the optical flow of adjacent video frames, a methodology to estimate the human density from single video frames, and procedures to geo-reference the information to be able to calculate the human pressure as a metric measure.

Besides deriving the human density from images, one might think of other applications, like calculating a chicken density to monitor if those animals have enough space according to the current regulations in livestock farming. Alternatively, estimating the density of olive trees from airborne images to monitor their water supply. And obviously to measure the number of cars on the highway via a car density estimate.

The best performing approaches for density estimation from single images are building on deep learning, in particular, on deep convolutional neural networks. Thus, the main scientific interest is to introduce and explore novel network architectures and to evaluate them on existing and new data sets. However, these evaluations are often not generalizing very well, as the predefined evaluation metrics and test protocols are suffering from several limitations, making comparisons of different approaches difficult. Thus, the goal of this paper is to intensify these limitations and to show how we can mitigate them.

In particular, as most important critical aspects we identified: (1) Ambiguities and inaccuracies in ground truth generation propagate to the resulting accuracy of the density estimate; (2) State-of-the-art databases are biased and thus limit the applicability of the trained models for real-life applications; and (3) The evaluation metric used for density estimation is not directly related to the loss functions used in current methods. To illustrate this, we implemented a baseline framework building on [9] and successively added enhancements, which have then be run on well-known benchmark datasets. The experimental results show that the proposed enhancements allow higher accurate density estimation and thus outperform the baseline approach. Additionally, in this way, we are able to highlight limitations in the test and evaluation protocol, which again enables more accurate density estimation and thus person counting.

This paper is structured as follows: Section 1 introduces the topic of density estimation from images and presents the main aim of the study. Next, the state-of-the-art is reviewed in Section 2. Then, we review the method described in [9] in detail, which can be considered our baseline, in Section 3. We choose this particular method since the underlying network is rather simple and adequate to show the general limitations of a multitude of recently published methods. Then, the core contribution of this work is presented in Section 4, where critical aspects and limitation are identified, presented, and discussed. Here, we analyze different aspects such as ground truth generation, evaluation and loss metrics, data augmentation, hyperparameters and training, and physical plausibility which reach beyond state-of-the-art and thus presents novel work by the authors. Our own implementation of the algorithm of interest is briefly presented in Section 5. Next, results are reported in Section 6, followed by a list of recommendations in Section 7 to overcome the presented critical aspects. Lastly, concluding remarks are given in Section 8.

## 2. Related Work

In the following, we give an overview of related work. First, we describe the main concepts for counting objects in images. Then, we discuss specific state-of-the-art methods based on convolutional neural networks (CNNs) in detail.

### 2.1. Concepts of Counting

Following [3,9,10], there are three different concepts of person counting in images (or object counting in general): counting (a) by detection, (b) by regression, and (c) by density estimation.

#### 2.1.1. Counting by Detection

Counting by detection is the most intuitive way for object counting and, thus, the way humans count. The concept is to detect each object instance in the image and thereby collecting the overall object count. However, robust object detection is a hard task in computer vision and not reliably solved [11,12]. Especially, when objects get smaller and overlap with each other, reliable detection of all instances cannot be guaranteed. In addition, detection is a harder problem than counting. Examples of counting by detection are [13,14].

#### 2.1.2. Counting by Regression

To avoid detection, regression-based approaches try to find a mapping from various image features to the number of objects by using supervised machine learning methods. However, such methods do not use the location of the objects in the image instead they just find the regression to a single number, that is, the number of objects. Therefore, huge training datasets are necessary to achieve useful results. For instance, [15] apply background modeling to extract moving objects from video frames. Local features are then extracted from these regions and applied to train a neural network in a supervised manner that finally predicts the object count. Reference [16] used self-organizing maps for predicting the crowd level based on gray level dependence matrices. Another example is [17] where, after background subtraction, edge features are fed into a neural network to estimate the crowd level.

#### 2.1.3. Counting by Density Estimation

In contrast to the previous approaches, the main concept is to estimate the object density function whose integral over any image region holds the count of objects within this region. In the field of traditional computer vision, the methods in [10,18] yield the best results, mainly due to the clever defined *Maximum Excess over SubArrays (MESA)* distance metric. The main concept is to derive dense image features, like dense scale-invariant feature transform (SIFT) descriptors [19] or dense histograms of oriented gradients (HoG) [20], and relate them via regression to the ground truth density. This paradigm was, for example, applied in [8], where a custom-tailored HoG-based detector was defined that yields robust detection also under viewpoint variations. Reference [21] presented a novel training strategy based on random regression forest such that the computational effort in the training phase could be significantly decreased. Today, many CNN-based approaches exist that also perform counting via density estimation. The main difficulty is that objects occur at different scales which causes over or underestimation of the true object count. In [22] this issue is addressed by a switching network that first classifies the image patch under consideration in three different scale categories. Then, for each scale, a different CNN extracts the density map. The method in [23] is comparable to [24] as it uses a multi-column network to tackle the scale issue. The final density map is derived at full image resolution by using a set of transposed convolutions. In [25], several loss functions are combined (composition loss) to account for scale, resulting in a rather difficult network. In [26], a U-Net-based structure is proposed that is also evaluated on a novel aerial dataset. Four selected approaches [9,24,27,28] are described in more detail in the following section and in Section 3.

### 2.2. CNN-Based Methods for Counting

In the following, we give an overview of the most related CNN-based approaches in more detail. All of them use the counting per density estimation approach, however, with different network designs. For fine-tuning the hyperparameters, recent methods based on swarm intelligence (e.g., [29,30]) or based on genetic algorithms [31] can be applied.

#### 2.2.1. Multi Column Method

**Figure 3:**
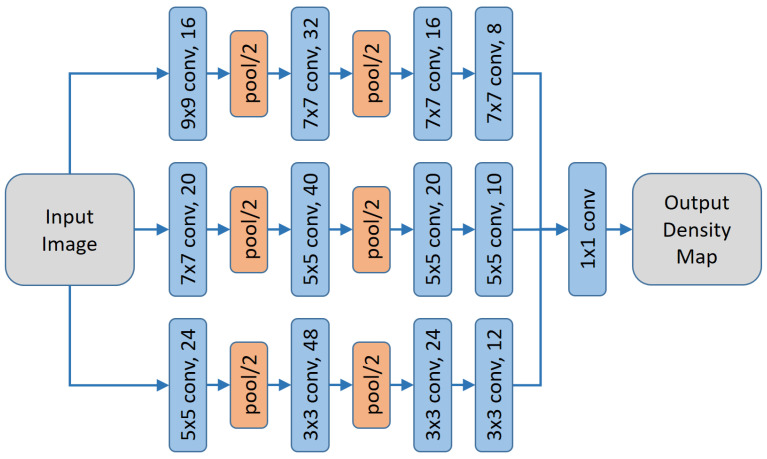
Multi column convolutional neural network (CNN) for person counting [24].

The Multi Column Method (MCNN) [24] is based on the idea that objects occur at different scales such that multiple parallel networks have to be combined to handle this multi-scale aspect. Therefore, the architecture consists of three parallel networks with different sizes of convolution kernels and thus varying receptive fields or scales. The resulting convolution layers of each CNN then produce the so-called merged feature maps that result in the density output (see Figure 3 for the network architecture). Training of such a multi column network is not trivial, especially, when only a few training images are available. Authors propose to train each column (i.e., one CNN) separately, before training the whole network. Such an iterative training method could also be performed for other network architectures and will be discussed in Section 4.5. In addition, [24] also introduced two crowd counting datasets, namely the *ShanghaiTech Part A* and *ShanghaiTech Part B* sets (Section 4.1). The head positions in all 1198 images were manually labeled and, as we will see later, the annotations also contain errors. Next, this work also introduces a method to generate the ground truth densities used for training. Their geometry adaptive kernel method is described in Section 4.2.

#### 2.2.2. Contextual Pyramid Method

**Figure 4:**
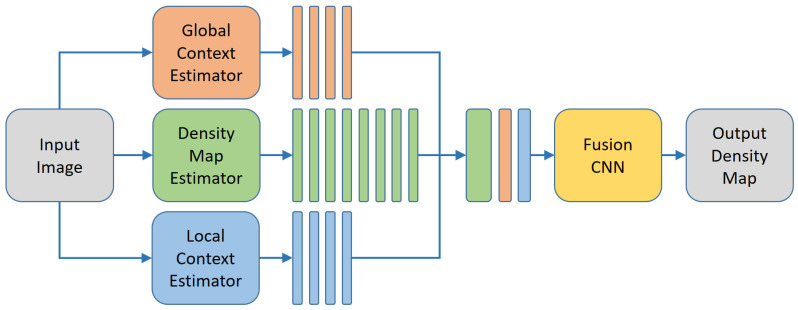
Contextual pyramid CNN for person counting [27].

Contextual Pyramid Method (CP-CNN) [27] builds on the observation that the MCNN approach yields deterministic biases w.r.t. the density within the given input image. The counting tends to overshoot for images holding only a few persons, at the same time to undershoot for images with dense crowds. Therefore, they added two more networks that should estimate the global and local context. Actually, there are many definitions of visual context, for example, those in [32,33]. The context for CNN-based density learning mostly refers to object scales resulting from perspective image acquisition. To this end, they associate a global context with the level of density in the given image. The density is classified into five classes, namely extremely low-density, low-density, medium-density, high-density, and extremely high-density. For learning this kind of contextual information VGG-16 [34] is used as feature extractor, in particular the first 13 convolutional layers combined with 5 max-pooling steps. Similarly, local context is also defined as the density level, however, on a local scale. To this end, the input for a rather small CNN (in terms of its parameters) are patches with a size of 64×64 pixels. This information is then fused with an MCNN network to undo the detected bias. The final fusion CNN is again a deep architecture, such that the authors avoid an end-to-end training of the overall framework. Instead, the two context estimator networks are pre-trained separately. Then, the density estimator and fusion CNNs are trained, by fixing the parameters of the context estimators. For more details, we would like to refer to [27]. Figure 4 depicts the network architecture, consisting of five parallel CNNs, which outputs are then jointly used in the fusion CNN as input.

#### 2.2.3. Context-Aware Method

Similar to the motivation from the CP-CNN method the core concept of the Context-Aware Method (CAN) [28] is to exploit contextual information during density estimation. Within this work, context is defined as a geometric property thus relating objects with their scale in the image. In contrast to MCNN and CP-CNN the CAN architecture is one straight network and therefore easier to train. Again, the first ten layers of VGG-16 are used as feature extractor. The authors claim that the features of VGG-16 encode the same receptive field over the entire image. To remedy this issue, they compute scale-aware features by performing spatial pyramid pooling [35] to extract multi-scale context information from VGG features. Those features are simple defined as the average over blocks of the VGG with varying block sizes k∈{1,2,3,6} that are then bilinearly upsampled to the original resolution to form the so-called contrast features. Those contrast features are then employed to learn weights for the scale-aware context features. The backbone, actually the same architecture as in [9], then produces the final density map. The proposed network yields good results and is an end-to-end trainable deep architecture. Nevertheless, it was not chosen as our baseline method, due to the increased model’s complexity in comparison to [9].

## 3. Baseline Method–Congested Scene Recognition Network

In this section, we discuss the Congested Scene Recognition Network (CSRNet) [9] in more detail, building the baseline for our approach. In this way, we put emphasis on the architecture of the CNN, the training stage, and the evaluation criteria. Many presented aspects also hold for various other recently published networks.

### 3.1. Architecture

The underlying idea of the network architecture is to take an existing and well-trained CNN as a deep feature extractor and add some new convolutional layers that will adapt to the density estimation task. This results in a single strait forward convolutional neural network. As in many other works (e.g., [27,28,36]) the widely-used VGG-16 architecture [34] is used as feature extractor.

**Figure 5:**
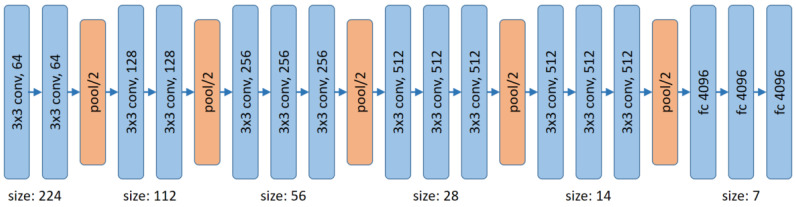
Architecture of VGG-16 [34].

The architecture of VGG-16 is depicted in Figure 5. The main concept is to use only 3×3 kernels in the convolutional layers, 2×2 max-pooling, and fully connected layers at the end. Overall, the authors in [34] argue that a deeper network with smaller kernels performs better than a shallow net with larger kernels.

**Figure 6:**
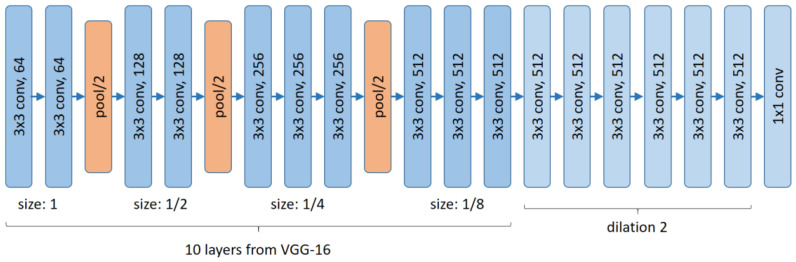
Architecture of Congested Scene Recognition Network (CSRNet) [9].

The idea of CSRNet [9] is to take the first 10 convolutional layers and 3 max-pooling layers with stride 2 from VGG-16 as feature extractor and to add 6 additional convolutional layers with rectified linear units (ReLUs) as activation functions to encode the actual task (backbone, cf. Figure 6). Note that the feature extractor consists of about 7.6 million parameters, while the backbone has about 8.6 million (7,635,264 and 8,628,225 to be precise). By first intuition, the backbone with roughly the same amount of parameters as the feature extractor is very large.

**Figure 7:**
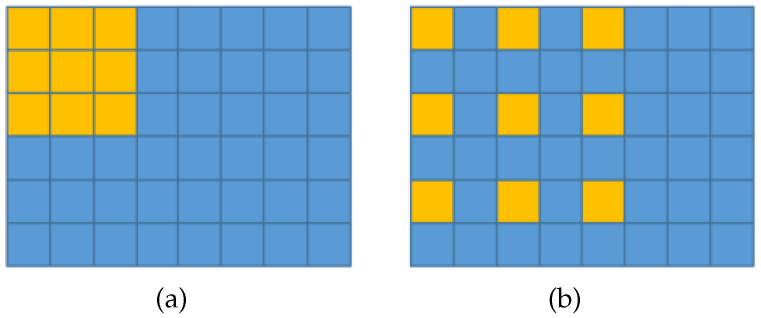
Concept of dilated convolution. (**a**) Dilation 1 and (**b**) dilation 2 are shown for a kernel of size 3×3.

The architecture of the CSRNet is depicted in Figure 6. In addition, the convolution layers in the backbone are based on dilated convolution (cf. Figure 7), where the authors argue that dilated convolutions enlarge the receptive field and thus can retrieve better results (cf. the definition of visual context in Section 2). This simple idea of dilation in the backbone is also used, for example, in [28].

Overall, CSRNet takes an RGB image of arbitrary size as input and predicts the human density at eighth resolution (due to the three 2×2 max-pooling layers). In contrast to multi-column architectures (MCNN, CP-CNN, or CAN), CSRNet is a simple network that can easily be trained end-to-end.

### 3.2. Training

To train a network, several aspects have to be taken into account: ground truth generation, data augmentation, design of the loss function, overall training strategy, and finally the specific hyperparameter settings.

Ground truth generation is discussed later in Section 4.2. For data augmentation, several options do exist. For example, [9] extract nine patches from each image at different locations with one-quarter of the original image size. The first four patches contain the four quarters of the image without overlap while the other five are randomly cropped from the image. Note that those patches are of different sizes for each image (as the dataset contains images of arbitrary dimensions) such that the network could only be trained with a batch size of 1. Additionally, in the source code, this augmentation is not used with the argument that the resulting accuracy is lower without any cropping of patches. However, without augmentation, the datasets are really small. For instance, the *ShanghaiTech Part A* set then only holds 300 training images, where over-fitting easily becomes an issue. We present a more general augmentation method in Section 4.4. Most current works use a pixel-wise Euclidean loss function, for example, [9,22,24,26,28]. Others use combined losses, for example, Euclidean with a structural similarity index measure (SSIM) loss [23] or with adversarial loss [25]. The pixel-wise Euclidean loss function could be simply defined as

(2) $$L\left(\Theta \right)=\frac{1}{2N}\sum_{i=1}^{N}{∥Z\left(X_{i};\Theta \right)-Z_{i}^{gt}∥}_{2}^{2}\;,$$

where L(Θ) is the loss function, *N* the batch size, $Z(X_{i};\Theta )$ the predicted density from image $X_{i}$ generated by the net with parameters Θ, and $Z_{i}^{gt}$ the ground truth density of image $X_{i}$. As an overall training strategy, one could first fix the feature extractor and only train the backbone, while training the whole network in a second step. Alternatively, the complete network could be trained end-to-end, as proposed in [9]. Finally, specific settings are of importance like the optimization algorithm such as stochastic gradient descent (SGD) algorithm and related hyperparameters like the learning rate. Our baseline uses SGD with a learning rate of 1 × 10${}^{-6}$.

### 3.3. Evaluation

Most recent work is based on the standard evaluation strategy (cf. [3,10,24,37]), where parts will be described in Section 4.3. As metrics, the mean absolute error (MAE) and the root mean squared error (RMSE) over all images are defined, with

(3) $$\mathrm{MAE}=\frac{1}{N}\sum_{i=1}^{N}\left|C_{i}-C_{i}^{gt}\right|\;\;\mathrm{and}\;\;\mathrm{RMSE}={\left[\frac{1}{N}\sum_{i=1}^{N}{\left(C_{i}-C_{i}^{gt}\right)}^{2}\right]}^{\frac{1}{2}}\;,$$

where *N* is the number of images, $C_{i}^{gt}$ the ground truth count for the image number *i* and $C_{i}$ the predicted count using the network. Overall, the evaluation is just based on the person count per image and not on the distribution of the according to density estimate.

## 4. Discussion of Critical Aspects

As core contribution of this paper, we identify and discuss many critical aspects that are influencing the resulting quality of crowd counting.

### 4.1. Datasets

**Figure 8:**
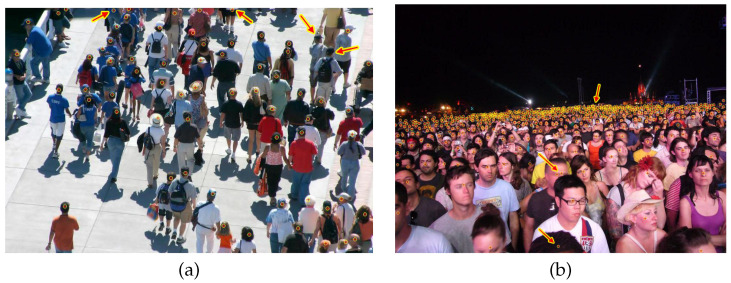
Two images from the ShanghaiTech Part A training set overlaid with the ground truth head annotations (yellow circles with red crosses inside). (**a**) Some annotations are missing and in the upper part there are torsos annotated instead of heads. (**b**) Double annotation and a very inaccurate annotation in the front. Additionally, with increasing distance from the camera the crowd becomes very dense. There, at some arbitrary point in the distance, the labeling stops.

Currently, there are some datasets available that are very useful for training and for evaluating crowd counting algorithms. Table 1 gives an overview of the most important specification of the most used crowd counting datasets. Figure 8 depicts two images from the ShanghaiTech Part A test set overlaid by the ground truth head annotation. Problems in terms of missing and sloppy annotations are superimposed with red arrows with yellow outlines. Any incorrect ground truth limits the potential of any machine learning algorithm. Besides this accuracy aspect, most datasets hold only a few images or are of very coarse resolution. In the future, the largest sets should be used which are currently the UCF-QNRF and the NWPU-Crowd datasets. The authors of [25,38] state that after labeling a correction step was performed by people that did not take part in the initial annotation process. Additionally, those datasets offer larger images, such that there is more potential for different data augmentation strategies. Due to comparability, basically all recent works compete just on the different datasets but do not perform cross-validation between those datasets. This limits the use for real-life applications as algorithms are more or less designed to just perform decently on the specific dataset, even if the results would just be based on over-fitting. This issue is well known in other domain such as in object recognition [39] or in mono-depth estimation [40], and should be considered.

### 4.2. Ground Truth Generation

In all current datasets, the reference annotation is given as coordinates of human heads in the images [24,25,26,37,41,42]. Thus, the density function has to be derived from these head coordinates. One simple version of density construction is to start with an image initialized with zeros. Then, for each (rounded) integer head location the density image is incremented by one. This result is then blurred with a Gaussian kernel with a fixed defined sigma. This strategy is often used, for example, in [3,8,10]. However, as humans appear at different scales it might be a good idea to adjust the Gaussian sigma with the object’s scale. In this case, the density function generation can be formulated as follows (formulation adapted from [43]): Given is a collection of 2D points $P_{i}^{gt}=\left\{P_{1},P_{2},\ldots ,P_{n}\right\}$ indicating the ground truth head positions in an image *I*. The ground truth density map $D^{gt}$ of *I* is obtained by convolving annotated points with a Gaussian kernel $N(p,\mu ,\sigma^{2})$. Thus, the density at a specific pixel *p* of *I* could be obtained by considering the effects from the Gaussian functions centered at all annotation points, potentially with varying $\sigma_{j}$:

(4) $$\forall p\in I,D^{gt}\left(p|I\right)=\sum_{j=1}^{n}N(p,\mu =P_{j},\sigma_{j}^{2})\;.$$

**Figure 9:**
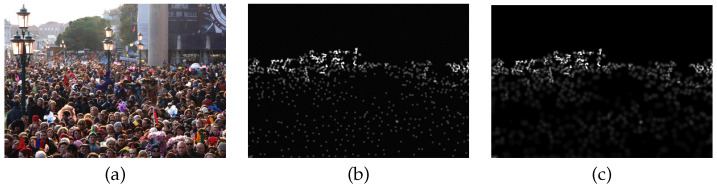
The density maps for the image (**a**) shown with a fixed sigma (**b**), and with the geometry adaptive sigma (**c**).

The authors of [24] argue that in standard images the size of persons varies with the distance to the camera (due to perspective geometry). According to that, humans in far distance should be represented as Gaussian blobs with smaller sigma and vice versa. However, as the geometry is not known an empirical method is chosen. The underlying idea builds upon the observation that people are closer to each other (in terms of pixel distances) if they are smaller in the image. Therefore, a method is proposed that takes the average distance to the k-nearest neighbors to calculate the sigma for the current annotation:

(5) $$\sigma_{i}=\beta d_{i}\;,$$

with $d_{i}$ the average distance of the three nearest neighbors of annotation *i* and β=0.3. Figure 9 depicts the differences of a fixed sigma (b) and the geometry adaptive sigma (c).

As different implementations produce the density at different resolutions (e.g., due to max-pooling layers) the density maps have to be accordingly downscaled for training. For example, the reduction from input to output size is of factor 4 for MCNN [24] and 8 for CSRNet [9] in both image dimensions. The naive approach is to rescale the image by this factor and then multiply each pixel by the squared factor. However, if, for instance, cubic convolution is used as an interpolation function then the sum over our density changes significantly. Therefore, a fixed protocol should be defined as how the ground truth density should be generated also keeping different resolutions in mind. In our simple implementation, the ground truth densities are generated at the resolution that the CNN outputs. Then the generation of ground truth and thus the evaluation defined in Equation (4) could be speeded-up significantly.

**Figure 10:**
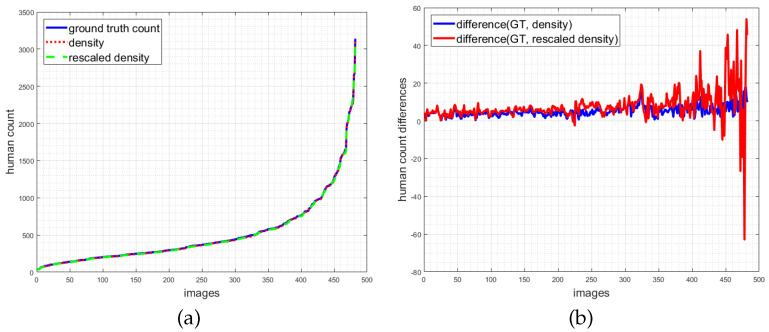
Count errors introduced during preprocessing. (**a**) Human count for all images based on ground truth head annotations, density estimate and downscaled density estimate. (**b**) Differences of ground truth head annotations and density estimates (blue), and differences of ground truth head annotations and downscaled density estimates (red).

For illustration, Figure 10 visualizes two types of count deviations. Figure 10a depicts the sorted human counts for all images of the ShanghaiTech Part A set (training and test set). Shown are the number of ground truth head annotations, the integral over the density determined with the geometry adaptive kernel, and the density downsampled by a factor of 8 used during training. Even though the counts look identical, they are actually not, which is depicted in the adjacent difference plots (Figure 10b). They show the differences of head annotation count to the two densities. It is well visible that the blue graph, representing the difference to the density at the original scale, is always above zero. Thus, the density has a lower human count w.r.t. the ground truth. The reason is that blurring of the annotations does not sum up to 1 as parts of the Gaussian kernel may be located outside of the image (also discussed in Section 4.3). The red graph depicts the difference in the downsampled density based on bicubic interpolation which, as discussed, alters the sum. While the blue and red curves are quite similar for lower counts, they deviate significantly for larger crowd counts. The largest absolute deviation is actually in the range of 63 humans. These inaccuracies have to be taken into account when judging the resulting MAE numbers.

Overall, the protocol for generating density maps from ground truth annotations as defined in previous works (e.g., [9,25,28]) is not optimal. Quite likely, by defining a more coherent ground truth density estimate, the training process and, thus, its results would gain accuracy.

### 4.3. Evaluation

The MAE and RMSE metrics per image as given in Equation (3) were initially defined for counting, not taking density estimation into account. Historically, these measures were defined for simpler datatsets where all images were of identical size and where also the object sizes and counts per image did not change too much. Such an assumption, for example, holds for the VGG Cell Dataset presented in [10]. However, person counting datasets include images of various resolutions and highly varying densities. Thus, larger images with more pixels automatically produce a larger MAE than smaller images (the same holds for the RMSE metric). To get a well defined unbiased metric we propose to define the pixel-wise MAE or at least normalize it, for example, w.r.t. the count as proposed in [26]. In addition, such that this novel metric is easier to interpret we propose to normalize this metric for specific sizes of images.

**Figure 11:**
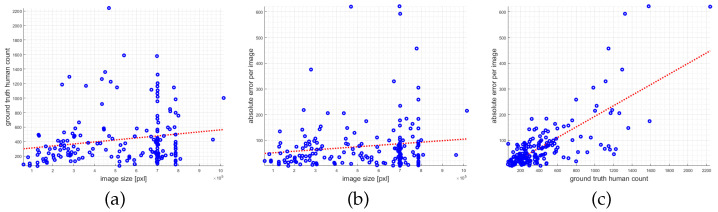
Scattergrams of (**a**) image size versus ground truth human count, (**b**) image size versus absolute prediction error, and (**c**) ground truth count versus absolute prediction error for the ShanghaiTech Part A dataset. The red dashed lines show a polynomial regression of degree 1 based on least-squares fitting.

To emphasize the discrepancies with the MAE measure three scattergrams are depicted in Figure 11. The scattergram in (a) plots the image size (total number of pixels) versus the ground truth human count. Here, a trend is visible, that larger images also contain more humans, which biases the MAE measure. The scattergram (b) shows the relation of the image size to the absolute count error of one of our CNNs. Also here a trend is observable that for larger images a larger error is produced (this relates to the previous plot). Finally, in (c) the ground truth human count is plotted versus the absolute count prediction error. Here, a linear trend is observable, that in the case of denser crowds also the counting error becomes larger. While the last aspect is logical, the first two indicate biases in the dataset.

Another critical aspect is the fact that the pixel-wise MAE reported during training does not correspond with (i.e., are not linearly correlated to) the image-wise MAE as defined in Equation (3). This is obviously the case for most published methods since in training the pixel-wise Euclidean loss is optimized (cf. Equation (2)) which is a measure per pixel. Consequently, the network optimizes a loss function that does not correspond to the metric in which the evaluation is performed. This is also the case why a better model (better w.r.t. the pixel-wise Euclidean loss) does not automatically result in a lower image-wise MAE. Therefore, when always taking the best model after, for instance, 1000 epochs there is a high variation in the image-wise MAE. This important aspect never has been discussed in the literature so far, even though all methods optimize a loss function that does not correspond to the evaluation function (cf. [9,22,23,24,25,26,28] and in particular Section 3.2). Since current works compete only with image-wise MAE and RMSE measures it is questionable which of the proposed network architectures really yield the quantitative best density in a real world scenario. In combination with over-fitting on small datasets like the ShanghaiTech Part A current benchmark results have limited informative value.

**Figure 12:**
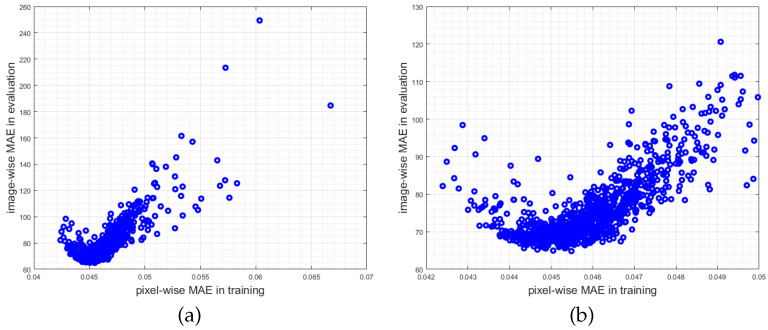
Scattergrams of pixel-wise mean absolute error (MAE) during training with resulting image-wise MAE in evaluation. (**a**) Scattergram for the complete data. (**b**) Scattergram only showing pixel-wise MAEs during training with values below 0.05, such that this plot is a zoomed version of (**a**). A non-linear correlation between both measures in visible.

With the knowledge gathered above, it is rather easy to outperform the results of our baseline method [9], by not taking the model with the lowest pixel-wise MAE in training, but the one with the lowest image-wise MAE. Figure 12 shows an example where the pixel-wise MAE during training is plotted versus the image-wise MAE during the evaluation on the test set (cf. Equation (3)) over 1000 epochs of fine-tuning. It is also very likely, that an area normalized MAE (or area normalized Euclidean error) defined as our loss function instead of Equation (2), would lower the image-wise MAE (cf. Equation (3)), which again also holds for all other state-of-the-art methods discussed in Section 2. However, the resulting density estimates would get worse.

Another issue has been found in the definition of the calculation of MAE and RMSE (cf. Equation (3)). It is claimed that the predicted person count $C_{i}$ is compared to the ground truth count $C_{i}^{gt}$. However, in reality (e.g., in the published source codes on GitHub (https://github.com/leeyeehoo/CSRNet-pytorch)) the $C_{i}^{gt}$ is determined as the sum over the downsampled density map (cf. previous section). Therefore, any deviation in the ground truth density map generation changes the reference person counts and thus the evaluation. For example, if each ground truth density value is divided by a factor of two also the MAE would be halved (even though this novel network would give incorrect predictions). This aspect is particularly an issue for head annotation on the image border. There a part of the Gaussian kernel is located outside of the image borders, such that the sum over the density is always smaller than the integer annotation input count. Especially, large objects on border change the *ground truth* count significantly, as shown in Figure 10.

Overall, for a fair comparison there should be a protocol that all participants have to follow. In the best case with a reference implementation. Similar to famous benchmarks as the Middlebury set for image matching (http://vision.middlebury.edu/stereo/) [44] or the Cityscape dataset for semantic segmentation (https://www.cityscapes-dataset.com/) [45].

### 4.4. Data Augmentation

Data augmentation is an important task to get robust results and to avoid over-fitting. Therefore, we suggest using to following augmentation strategy. Each training image has a 50% chance to get augmented at each epoch. The following warp options are set:

- Rotations up to 10 degrees.
- Shifts in width and height of 2% of image dimensions.
- Zoom range of 1%.
- Horizontal flipping.
- Radiometric offset and gain of 1% for each image channel.

Note that the zoom range should be small or even disallowed as zooming of the target density map changes the integral over the resampled new density and thus also of the human count. If zooming is explicitly wanted then this function has to be discretely implemented such that after resampling the density map is radiometrically correctly rescaled. Now, in contrast to our baseline where only the original images are used at each epoch, our training tends to less over-fitting.

### 4.5. Hyperparameters and Training

In all our experiments the *Adam optimizer* [46] is used with a fixed learning rate of 1 × 10${}^{-6}$, due to better convergence in comparison to the classical SGD algorithm. The batch size is set to 1 in all experiments, using the whole images. This reduces the density cropping effects on the image border and results in higher accuracy (i.e., lower MAE over the dataset). The VGG-16 layers of the network are pre-trained on ImageNet [47].

Since the VGG-based feature extractor is already fine-tuned its parameters may be fixed in the first training step. By doing so, only the backbone can be trained, for instance, with a larger learning rate. Experiments showed that in the case of little data augmentation, this iterative training is necessary to achieve convergence. With stronger data augmentation the whole network can be trained at once, simplifying the training procedure.

### 4.6. Physical Plausibility

Even though an object density cannot be negative, this fact is not encoded in some models, such as the baseline model. While other methods for density estimation enforce non-negative outputs [3,8,10,18], the presented CSRNet does not. Thus, there are negative density values in the result. In the future, it would be beneficial to constrain the CNN output to non-negative density values. One simple solution would be to add a ReLU function to the output layer. Obviously, the training would take more time to converge but physical plausibility could be accomplished.

## 5. Implementation Details

Our implementation is based on the Keras version on GitHub (https://github.com/Neerajj9/CSRNet-keras) which is an unofficial Keras/TensorFlow port of the PyTorch version on GitHub (https://github.com/leeyeehoo/CSRNet-pytorch) [9]. We support multiple graphical processing units (GPUs) in the inference and in the training if batch sizes larger than 1 are employed. However, the best quantitative performance has been achieved by using the whole images as training input, which is consistent with the observations reported in [9]. The only downside of this approach is, that since training images have different dimensions, a batch size of 1 has to be used, leading to the usage of only one GPU during training.

## 6. Results

This section first report on results based on the ShanghaiTech Part A dataset and second on real-world videos.

### 6.1. ShanghaiTech Part A Dataset

Table 2 depicts the MAE values over the ShanghaiTech Part A dataset for different dilation rates as reported in [9]. According to these numbers, a dilation rate of 2 boosts the results in comparison to a dilation rate of 1 by a 1.5 MAE change. Since the MAE values fluctuate very much at each epoch it is to be questioned if this boost is of significance or just based on coincidence.

**Figure 13:**
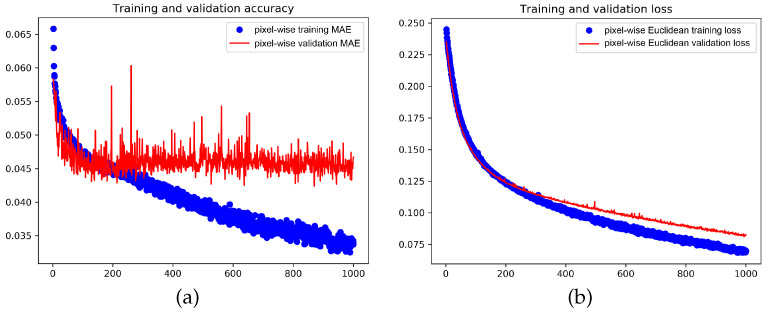
Learning and convergence rate, and loss of CSRNet with a dilation rate of 2 in the backbone. Here, all 16.2 million parameters are adjusted at once using the Adam optimizer. (**a**) The pixel-wise MAE is shown over 1000 epochs for the training set (blue) and the validation set (red). (**b**) The training and the validation Euclidean losses are shown over 1000 epochs.

Exemplary results from training the CSRNet are depicted in Figure 13, showing (a) learning and convergence rate, and (b) loss. Here all 16.2 million parameters of the baseline model are adjusted at once using the Adam optimizer. The graph in (a) gives the pixel-wise MAE on the training set over 1000 epochs, which gets lower and lower, and shows that the pixel-wise MAE on the validation set is noisy and flattens out, that is, converges, after 200 epochs. The Euclidean losses are given in (b) and they get lower and lower over the epochs.

**Figure 14:**
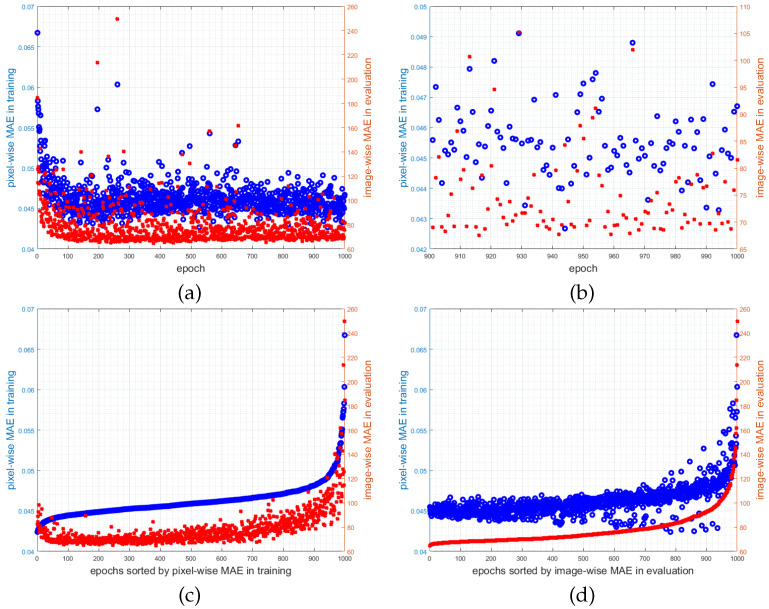
The graphs (**a**,**b**) plot the pixel-wise MAE used during training (blue) and the image-wise MAE used in evaluation (red). Note, that those two metrics do not correlate well. (**b**) Detailed view on the last 100 epochs. (**c**,**d**) These graphs show the same information, but first sorted by the pixel-wise MAE used during training and second by the image-wise MAE used in evaluation.

To emphasize the misalignment of the metrics, the pixel-wise MAE in training and the image-wise MAE in evaluation are plotted in Figure 14 into one plot using two y-axes with different scales (both based on the test set). In the plots (c–d) sorted by one of the two metrics, it is clearly visible, that by minimizing one metric the other one is not minimized. As stated in Section 4.3 the proposed CNN does not optimize the evaluation metric given in Equation (3). Same holds for all methods presented in Section 2.

**Figure 15:**
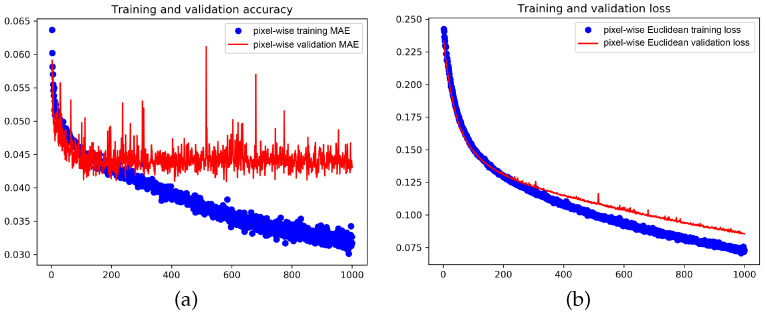
Learning and convergence rate, and loss of CSRNet with a dilation rate of 1 in the backbone (note that the plots in Figure 13 are based on a dilation rate of 2). Here, all 16.2 million parameters are adjusted at once using the Adam optimizer. (**a**) The pixel-wise MAE is shown over 1000 epochs for the training set (blue) and the validation set (red). (**b**) The training and the validation Euclidean losses are shown over 1000 epochs.

Since the authors of [9] claim that the dilation rate of 2 in the backbone is a very important design issue, we also trained a network with dilation 1. Plots are given in Figure 15. And indeed, the resulting accuracy with a dilation rate of 1 is lower than the one using 2.

Overall, the number of parameters in the backbone of the baseline model is very large (cf. Section 3), such that a smaller backbone should also be able to produce good density predictions. However, this has not been evaluated within this work and left for future work. Finally, the MAE values for different approaches are depicted in Table 3 together with the spatial reduction size factor of which the density map is predicted. Here, our version of the CSRNet outperforms MCNN already at epoch 6 and CP-CNN at epoch 10, which confirms a good performance of the VGG-16 as feature extractor. Additionally, by tweaking the training methodology we get a boost of 3.4 in the MAE metric w.r.t. the original paper [9] for dilation rate of 2, and 2.2 MAE for dilation rate of 1. Recall, that this boost is larger than authors report w.r.t. the dilate rate test reported in Table 2. Actually, the CAN network performs a bit better than our proposed method. However, we just enhanced the baseline method [9]. Incorporating our findings into the CAN network would also enhance its performance.

### 6.2. Real-World Videos

**Figure 16:**
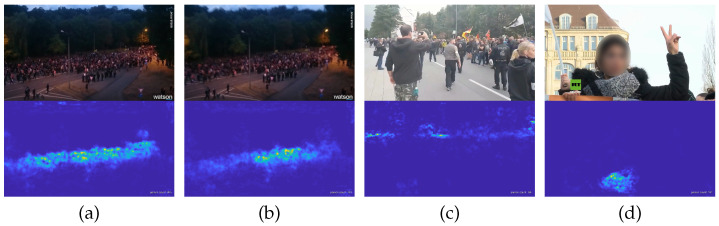
Exemplary results for three different demonstrations in Chemnitz and in Berlin from 2018 (original images from YouTube, urls given in the text). (**a**,**b**) Two consecutive video frames, where (**b**) is slightly blurred due to camera motion. While the person count is predicted with 205 for (**a**), it drops to 101 for the blurry image (**b**). (**c**) Person count prediction is 68 for this image. (**d**) The algorithm fails and classifies the texture of the scarf as a highly dense crowd, resulting in an incorrect prediction of 101 individuals.

Last but not least, our model with the best MAE was used to predict the human densities for all frames of three YouTube videos consisting of over 21,000 frames, with exemplary results shown in Figure 16. The videos were chosen from demonstrations in Berlin (https://www.youtube.com/watch?v=Ah7wZmrlaVE) and Chemnitz (https://www.youtube.com/watch?v=KYYlS-xLm2g, https://www.youtube.com/watch?v=KUv3moibKzY) in Germany. As expected the network yields strange incorrect predictions on images that were not part of the training set (cf. Figure 16d). For instance, images not holding any person were not used in training and result in arbitrary predictions. Here we see, that a larger dataset is needed for training. Coarse and dense crowds but also images not holding any person should be part of the training data. Conspicuously, blurred images yield incorrect count predictions (cf. Figure 16b). Without going into more detail it seems obvious that smaller errors in some scientific benchmarks do not automatically correspond to usability in real-life situations. This emphasizes our call for evaluations across datasets instead of just intra-dataset testing as argued in Section 4.1. Additionally, if the occurrence of a certain image degradation is known for the given final application, for example, blurring, then this degradation can be simulated within the data augmentation process (cf. Section 4.4).

## 7. Recommendations

Based on the findings within this work, we present several recommendations that allow for a fair comparison of different methods. In this way, the trained models are not limited to specific benchmarks but can be applied in the wild for different kinds of data.

### 7.1. Ground Truth Density Generation

The generation of ground truth densities has to be fixed for each dataset. Either by supporting the density for each image at full image resolution in a float-valued image format (e.g., TIFF) or by supplying a (*python*) source code for density estimation based on the point-wise or box-level annotations. For a fair comparison all methods have to compare their results to exactly those ground truth densities, which will eliminate all aspects discussed in Section 4.2.

### 7.2. Evaluation Metrics

To allow a more general evaluation, we propose three additional metrics: pixel-wise MAE, pixel-wise RMSE, and pixel-wise mean normal absolute error (MNAE). The MNAE is defined as (cf. [26,38], for description of variables see Section 4.3)

(6) $$\mathrm{MNAE}=\frac{1}{N}\sum_{i=1}^{N}\frac{\left|C_{i}-C_{i}^{gt}\right|}{C_{i}^{gt}}\;.$$

The normalization gives the relative errors (i.e., percentages) rather than the absolute counting errors, such that it better balances residuals of sparse and congested scenes. Since the quality of density estimation should be judged primarily, the most important measures are those pixel-wise metrics over the densities at full-resolution (pixel-wise RMSE is directly dependent to the Euclidean loss). Only as second metrics the image-wise MAE, RMSE, and MNAE should be evaluated, where the initial person count (i.e., the ground truth count) is not based on the number of annotations in the image, but on the integral over the supported density.

Please note, changing from image-wise to pixel-wise metrics will significantly alter the rankings of current methods. An example is given in Figure 2, where two images are shown both holding 254 people but with very different spatial density distribution. Just comparing the final human count does not satisfy the complexity of density estimation. Overall, those recommendations will fix the problems discussed in Section 4.3.

### 7.3. Fair and Unbiased Dataset

For a fair evaluation, the dataset should hold training, validation, and test set. Only the training and the validation set are given to the participants, while the test set is hidden on a server that performs the final evaluation. This fixes a lot of issues like different ground truth density estimates or fine tuning to the test set. Furthermore, negative images not holding any person should be part of the dataset. Additionally, multiple datasets should be used for evaluation to show if methods are able to generalize. For example, training on UCF-QNRF [25] and testing on NWPU-Crowd [38], and vice versa. An algorithm that performs well on unseen data will also perform better in the wild and, thus, lowering the probability of failing in real world application as discussed in Section 6.2. Biases in the datasets like observed in Section 4.3 should be avoided. One solution is to increase the number and diversity of image within a dataset and to acquire the data from multiple different sources (cf. [38]).

## 8. Conclusions

Our core contributions are the identification of the limitations of current state-of-the-art approaches for person counting and density estimation from single images and the provision of insights on how to mitigate these limitations. The most important critical aspects can be summarized as follows: (1) Ground truth generation is an important topic and any inaccuracies introduced in this step propagate to the resulting accuracy of the density estimation method. Ambiguities in ground truth generation were identified and discussed. (2) The image databases used for training and testing are biased and thus limiting the applicability of the trained models for real-life applications. (3) The metric used for evaluating density estimation algorithms is not directly related to the mainly used pixel-wise Euclidean loss function. Understanding this mismatching of evaluation metrics allowed to boost the accuracy of state-of-the-art methods. (4) A more optimized data augmentation increased the robustness of the training step. (5) Lastly, by applying the resulting insights to our own implementation we were able to outperform the results of our chosen baseline method [9] significantly. Furthermore, our insights and recommendations will help to advance the field of person density estimation in general by highlighting current evaluation protocol limitations and by proposing extensions.

The next steps are to discuss the issues within the research community and jointly define a unified evaluation protocol. Specific future work contemplated by the authors are as follows: (1) Comparison of existing methods based on the pixel-wise MAE, RMSE, and MNAE criteria to gain further insights. (2) Alteration of parameters of the baseline model, in particular to lower the number of convolutional layers in the backbone, and evaluation of its impact. (3) Constraining the baseline method to only generate non-negative densities to gain physical plausibility. (4) Performing a cross-validation based on the larger current datasets, that is, UCF-QNRF and NWPU-Crowd, to learn which method is generalizing well. (5) Switching from person counting to generic object counting in remotely sensed images by designing one network that predicts densities for multiple object categories at once.

## Figures and Tables

**Table 1:** Main specifications of crowd counting datasets. Datasets with varying image size are marked as (*).

Dataset	Number of Images	Number of Annotations	Average Count	Average Resolution	Publication
ShanghaiTech Part A	482	∼240 k	501	868 × 589 (*)	[24]
ShanghaiTech Part B	716	∼90 k	124	1024 × 768	[24]
UCF-CC-50	50	∼64 k	1280	2888 × 2101 (*)	[41]
WorldExpo10	3980	∼200 k	56	720 × 576	[37]
UCSD	2000	∼50 k	25	238 × 158	[42]
UCF-QNRF	1535	∼1250 k	815	2902 × 2013 (*)	[25]
NWPU-Crowd	5109	∼2130 k	418	3209 × 2191 (*)	[38]

**Table 2:** Estimation errors on the ShanghaiTech Part A dataset with different dilation strategies in the backbone. The numbers taken from [9].

Architecture	MAE
dilation 1	69.7
dilation 2	68.2
dilation 2 and 4	71.9
dilation 4	75.8

**Table 3:** Estimation errors on the ShanghaiTech Part A dataset sorted from largest to smallest MAE. We were able to boost the result of the baseline method [9] by 3.4 MAE without changing the network architecture. The spatial reduction size factor of the predicted density map is also given for each method.

Method	MAE	Size Factor
MCNN [24]	110.2	1/4
Switching CNN [22]	90.4	1/4
CP-CNN [27]	73.6	1/4
CSRNet [9]	68.2	1/8
SANet [23]	67.0	1/1
MRCNet [26]	66.2	1/1
**this work**	64.8	1/8
CAN [28]	62.3	1/1

## Abbreviations

The following abbreviations are used in this manuscript:

CAN	context-aware network
CNN	convolutional neural network
CP-CNN	contextual pyramid convolutional neural network
CSRNet	congested scene recognition network
GPU	graphical processing unit
HoG	histograms of oriented gradients
MAE	mean absolute error
MCNN	multi column neural network
MESA	maximum excess over subarrays
MNAE	mean normalized absolute error
RMSE	root mean squared error
MRCNet	multi-resolution crowd network
NWPU-Crowd	crowd dataset from Northwestern Polytechnical University
ReLU	rectified linear unit
RMSE	root mean squared error
SANet	scale aggregation network
SGD	stochastic gradient descent
SIFT	scale-invariant feature transform
SSIM	structural similarity index measure
UCF-QNRF	large crowd counting data set from University of Central Florida
UCF-CC-50	crowd counting data set from University of Central Florida
UCSD	crowd counting data set from University of California San Diego
VGG	very deep convolutional network from University of Oxford
